# Eye care practitioners and falls prevention for older adults: A scoping review

**DOI:** 10.1111/ggi.15098

**Published:** 2025-02-10

**Authors:** Jingyi Chen, Khyber Alam, Si Ye Lee, Anne‐Marie Hill

**Affiliations:** ^1^ Department of Optometry, School of Allied Health The University of Western Australia Perth Western Australia Australia; ^2^ WA Centre for Health and Aging The University of Western Australia Perth Western Australia Australia; ^3^ School of Allied Health The University of Western Australia Perth Western Australia Australia

**Keywords:** accidental falls, falls prevention, older adults, ophthalmology, optometry

## Abstract

**Aim:**

Eye care practitioners are well‐placed in the community to provide falls prevention advice to older adults, but existing literature offers scant insight into whether this occurs in practice. This scoping review aimed to map and synthesize the evidence for community eye care practitioners' awareness and behaviors in falls prevention in older adults, as well as barriers and enablers to implementation of falls evidence.

**Methods:**

This review process was guided by the Arksey and O'Malley framework for scoping reviews and PRISMA‐ScR guidelines. MEDLINE, CINAHL Complete, Embase, Web of Science and OpenMD were searched for published and gray literature between January 1990 to October 2024. Data were mapped against the World Falls Guidelines framework of: (i) risk stratification, (ii) assessment, and (iii) management, and a barriers and enablers framework.

**Results:**

A total of 19 sources met the inclusion criteria. Few studies directly captured results from eye care practitioners. The results suggested a gap in implementation of falls guideline evidence. Community eye care practitioners had low levels of awareness about falls, and were not routinely implementing falls history taking, risk stratification and assessment of contrast, visual fields, and stereopsis. Eye care practitioners might not be referring patients for exercise and environmental interventions.

**Conclusion:**

The evidence suggests that eye care practitioners have some awareness of falls prevention, but might benefit from better understanding of evidence‐based falls guidelines. There appeared to be gaps that exist between evidence and translation into practice. Future studies should explore practitioner experiences and implementation efforts to improve falls prevention in community eye care. **Geriatr Gerontol Int 2025; 25: 337–345**.

## Graphical abstract

A scoping review was carried out to map the evidence for eye care practitioners' awareness and behaviors in falls prevention for older adults. Results showed that eye care practitioners have some awareness of falls, but might benefit from better understanding and implementation of evidence‐based falls guidelines.

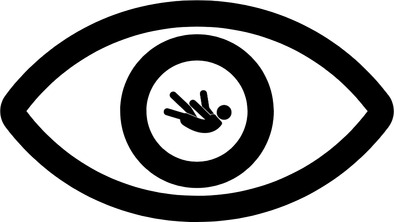

## Introduction

Falls are the leading cause of injury for adults aged >65 years,^1^ and often result in hospitalization, disability and increased risk of mortality.^2^ Annual medical costs attributed to falls are estimated to be US$50 billion in the USA, and A$2.3 billion in Australia.^3^, ^4^ With the aging population, fall‐related injuries are a serious and growing public health problem.^5^ Eye care practitioners can play an important role in falls prevention during their interactions with older adults.^6^ In many countries, a large proportion of the older adult population visit eye care practitioners for examinations.^7^, ^8^, ^9^ Older adults with visual impairment are almost twice as likely to fall compared with fully sighted individuals.^10^ Furthermore, patients who have fallen might present to an eye care practitioner for ocular trauma^11^ or damaged spectacles,^12^ which presents opportunities for the prevention of further falls.

Although there is compelling evidence to suggest that optimizing vision reduces the risk of falls,^13^ not all interventions might be beneficial. A randomized controlled trial studying eye care interventions found that the intervention group reported more falls compared with the control group.^14^ Probable explanations offered were that new spectacles require adaptation and might increase falls risk in the short term, or that vision improvements might have led to greater risk‐taking behaviors by participants.^14^ Progressive and bifocal spectacles are commonly prescribed for presbyopia, but evidence suggests that these can increase falls risk, and single vision spectacles should be worn outdoors instead.^15^, ^16^ Monovision contact lenses for presbyopia correction can also increase falls risk as they reduce stereoacuity.^17^

Although there is strong evidence that supports first and second eye cataract surgery for falls prevention,^18^, ^19^, ^20^, ^21^ a population‐based study found an increase in hospitalized falls after first‐eye cataract surgery.^22^ It was postulated that cataract surgery might have led to anisometropia and reduced stereopsis.^22^ The same authors reported an increase in hospitalized falls after second‐eye surgery, although to a lesser extent, suggesting that increased physical activity and risk taking might have contributed to this finding.^23^ The available range of evidence highlights that eye care practitioners must understand how specific interventions affect falls risk, and make careful recommendations to older adults in the community.

The World Falls Guidelines were published in 2022 as a resource for healthcare practitioners that synthesizes the available evidence on falls prevention.^13^ The guidelines recommend that all health professionals, including eye care practitioners, ask older adults about a history of falls as part of risk stratification.^13^ Older adults who are assessed to be at low risk of falls should be offered education about falls prevention and offered advice on exercise. If a patient presents with a fall‐related injury, they should be regarded as high risk for future falls. Recommendations specific to eye care practitioners include the measurement of visual acuity, depth perception, contrast sensitivity and other visual impairments, such as hemianopia. Management guidelines for visual impairment include cataract surgery, advice on adapting to new spectacles and advice on avoiding progressive spectacles outdoors. The guidelines encourage practitioners to refer older adults with severe visual impairment to occupational therapy for home environment assessment.

Falls prevention efforts by eye care practitioners can play an important part in reducing the number of falls and consequent injuries for older adults as part of a multidisciplinary approach.^13^, ^24^ However, there is a paucity of literature that have reported whether eye care practitioners are translating falls prevention evidence into practice. Therefore, the present scoping review aimed to map and synthesize the evidence for community eye care practitioners' awareness and implementation of falls prevention guidelines. This review aimed to identify gaps in current evidence and inform recommendations for future research.

## Methods

### Protocol

The methods for the present review are described in detail in a published protocol.^25^ A scoping review was considered appropriate, as researchers can incorporate a range of study designs in both published and gray literature, and address questions beyond intervention effectiveness.^26^ The review followed the Arksey and O'Malley framework for carrying out scoping reviews,^27^ and was reported in accordance with the Preferred Reporting Items for Systematic Reviews and Meta‐analyses extension for Scoping Review (PRISMA) extension for scoping reviews.^28^

### Research question

The broad research question that guided this study was “What is the evidence in current literature regarding whether community eye care practitioners are implementing evidence‐based practice for falls prevention?” Using the World Falls Guidelines^13^ as a framework, the objectives of this review were to identify the scope, nature and extent of evidence for community eye care practitioners: (i) performing fall risk stratification (ii) performing vision tests pertaining to falls risk, and (iii) recommending suitable management and intervention for falls prevention. Furthermore, this research aimed identify (iv) barriers and enablers to eye care practitioners implementing falls evidence in practice.

### Eligibility criteria

The inclusion and exclusion criteria were defined by ‘participants’, ‘concept’ and ‘context’.^27^, ^29^ The participants for this review were optometrists and ophthalmologists. The concept for this review was the role of eye care practitioners in translating falls prevention evidence into practice for older persons. This included awareness and knowledge of evidence, screening for falls risk, and adapting clinical assessment and management as recommended by evidence.^13^ The context was community eye care practices that operate in high‐income countries at primary, secondary or tertiary levels of care. All studies designs were included. Studies based in middle‐ or low‐income countries were not included, as different resourcing challenges apply; therefore, local context considerations are required for falls prevention recommendations in these countries.^13^ Specialized settings, such as low‐vision services, hospitals and university teaching clinics, were excluded. Studies were also excluded if they were published before 1990 to ensure recency of practice in accordance with current evidence for falls prevention^13^, ^24^ or if they were not published in English.

### Information sources and search strategy

The search strategy followed the three‐step process described by the Joanna Briggs Institute.^30^ An initial search of MEDLINE was undertaken to identify relevant articles. Identified keywords and index terms from these articles were mapped to MeSH headings, and used to develop a full search strategy with the guidance of an experienced librarian. The search strategy was adapted for each database searched, which were MEDLINE, Embase and CINAHL Complete. All study designs were included. Web of Science and the OpenMD Ophthalmology directory were searched for unpublished studies and gray literature. Reference lists of included studies were hand‐searched to identify further sources that were not part of the initial search yield. An example search strategy can be found in Supplementary File S1.

### Evidence selection

All identified citations from the search were uploaded to EndNote 20 and duplicates were removed. Two independent researchers (JC and KA) screened titles and abstracts. Full‐text articles were retrieved and assessed for inclusion based on the ‘participants’, ‘concept’ and ‘context’ criteria (JC and AMH). Any disagreements between reviewers were resolved through discussion, and a third reviewer (SYL) to arbitrate when needed.

### Data extraction and charting

A data table was developed by two reviewers (JC and AMH) to extract information that described eye care practitioners being aware of falls prevention evidence or undertaking fall prevention practice. Any inconsistencies between the two reviewers were discussed with a third reviewer to reach consensus.

### Data collation and reporting of results

Quotes were extracted verbatim and charted by two researchers (AMH, JC) according to the World Falls Guidelines framework^13^ under the subsections of: (i) falls risk stratification, (ii) assessment, and (iii) management and intervention. Data that identified barriers and enablers were collated separately. Consensus was reached on the data mapping and presentation of results by all authors.

## Results

**Figure 1:**
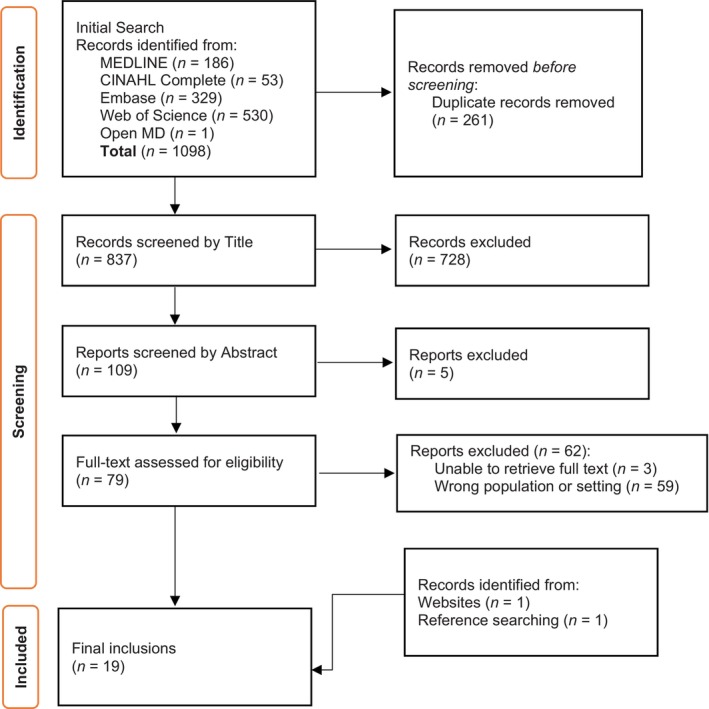
Preferred Reporting Items for Systematic Reviews and Meta‐analyses extension for Scoping Review flow diagram.

**Figure 2:**
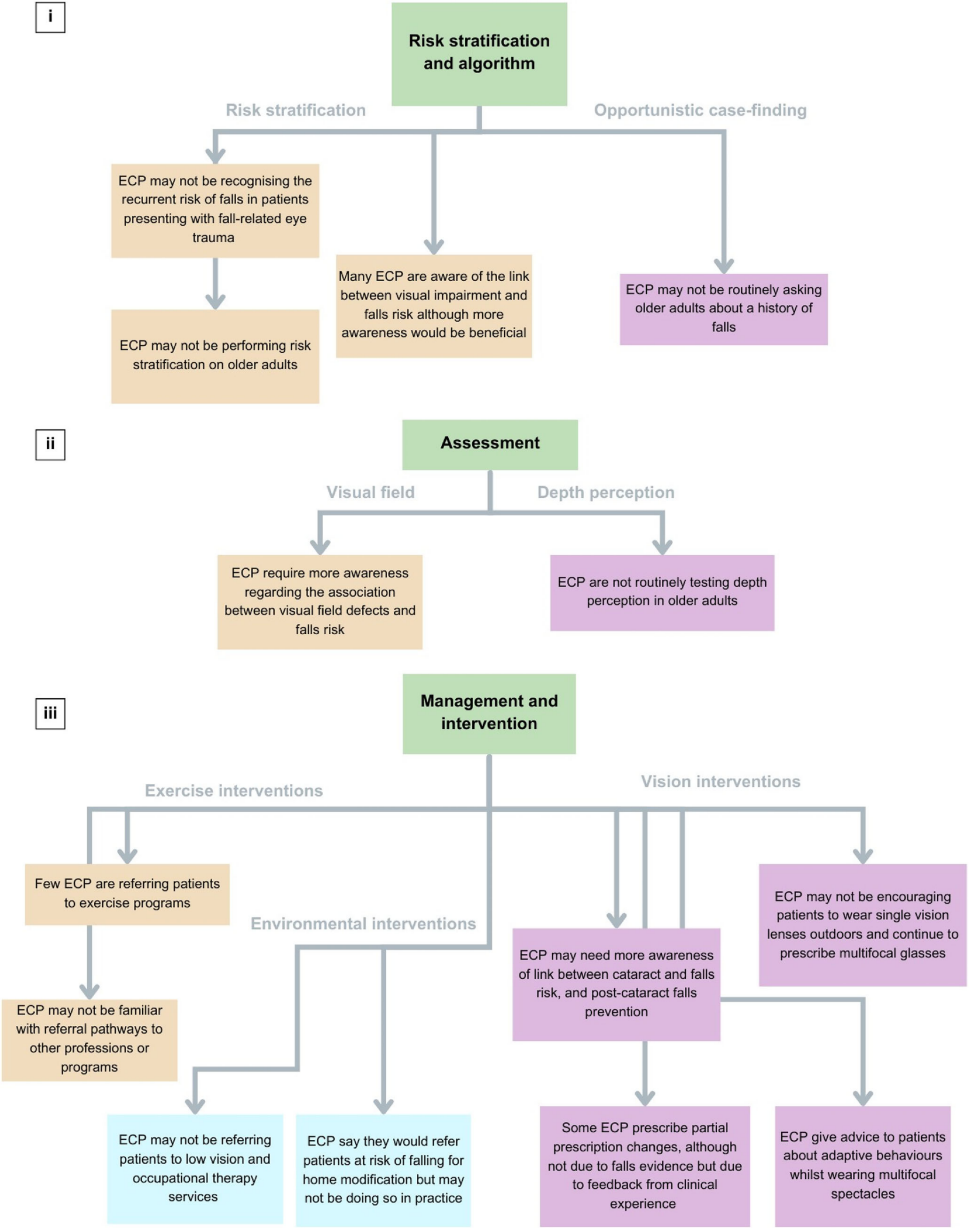
Eye care practitioner (ECP) falls prevention awareness and behaviors mapped against the World Falls Guidelines.^13^

The search of databases yielded 837 unique studies, of which 17 met the eligibility criteria and were included in the review (Fig. 1). One source was found on a website and one was found during reference list review, which contributed to a total of 19 included sources. Study characteristics are detailed in Table 1. Included sources were published between 2000 and 2024. Articles were from Australia (*n* = 6), the USA (*n* = 7), the UK (*n* = 5) and Italy (*n* = 1). Study designs included literature reviews, systematic review, retrospective and prospective cohort, Delphi, cross‐sectional studies, and case series (Table 1). Two sources of gray literature were identified, which was a report written by a university^31^ and an issue brief.^32^ Studies related to optometrists (*n* = 5), ophthalmologists (*n* = 6) and referred to both or broadly to eye care practitioners (*n* = 8). A summary of findings mapped against the World Falls Guidelines^13^ is shown in Fig. 2, and detailed data charting can be found in Supplementary File S2.

**Table 1:** Summary of included studies

Author, year	Country	Eye care practitioner	Study type	Summary and findings
Black & Wood, 2005	Australia	Optometrist	Review	Informs optometrists on evidence that links falls to visual impairment and suggests strategies for falls prevention.
Black *et al*., 2011	Australia	All	Prospective	Examined the predictors of falls in older adults with glaucoma. Falls occurred more commonly for community‐dwelling adults with more visual impairment, particularly the inferior visual field.
Boon *et al*., 2015	Australia	All	Prospective cohort	Investigated the perceptions of older adults with visual impairment on the causes of incidents, including falls, and prevention of future incidents. Participants had unmet needs from eye care consultations regarding advice on incident prevention.
Chang, 2021	USA	All	Review	Informs eye care practitioners about the evidence regarding monovision and multifocal spectacles, and increased risk of falls.
Dillon *et al*., 2022	Australia	Optometrist and ophthalmologist	Qualitative with deductive analysis	Investigated perspectives of stakeholders, including eye care practitioners, on fall prevention programs for older adults with vision impairment. Stakeholders considered falls prevention to be important, but lacked understanding on how to deliver this.
Elliott & Chapman, 2010	UK	All	Intervention	Changes in lens magnification influences step negotiation. Suggests that partial prescriptions should be given and that older adults should be warned of changes to ocular or spectacle magnification.
Elliott, 2014	USA	Optometrist	Review	Informs practitioners on how refractive correction changes, and spectacles can cause gait and postural changes, and lead to falls. Provides falls prevention recommendations to clinicians.
Evans & Rowlands, 2005	UK	Ophthalmologist	Review	Informs practitioners on the proportion of undetected visual impairment. Not all patients present for routine eye examinations, and even those diagnosed with eye disease fail to be referred to low‐vision services.
Garrigan *et al*., 2021	USA	Ophthalmologist	Systematic review	Explored the effect of age‐related macular degeneration on falls risk. Found that quality data was lacking. Recommended standardized assessments including falls risk stratification.
Ho *et al*., 2022	Australia	Optometrist	Delphi	Twelve optometry panelists contributed across two Delphi rounds. Most clinical recommendations for optometrists to implement falls prevention reached consensus, but some were identified to be less feasible to implement in real‐world settings.
Ho & Haddock, 2024	Australia	Optometrist and ophthalmologist	Issue brief	Summarizes challenges to optometrist and ophthalmologist roles in falls prevention, as well as subsequent policy recommendations.
Hill *et al*., 2001	UK	Optometrist	Report	Interviewed older people to better understand how they use stairs and assess safety. Some participants reported that they had been warned to be careful when using stairs when wearing bifocal spectacles and were provided advice, such as tucking their chin in when navigating steps.
Kavoussi *et al*., 2015	USA	Ophthalmologist	Case series	Identified indicators for prognosis for poor visual and anatomical outcomes for pseudophakic patients where they experienced fall‐related open globe injury. Summarized prognostic factors and concluded that ophthalmologists should contribute to falls risk discussions.
Mehta *et al*., 2021	UK	Ophthalmologist	Cross‐sectional	The rate of self‐reported falls by older adults in the previous 12 months at an ophthalmology department was lower than the reported prevalence in the general population. However, falls were not defined, so a participant might not have recognized a near‐miss, slip, trip or non‐injurious fall. A significant proportion of individuals who reported a fall wore single‐vision glasses.
Melillo *et al*., 2017	Italy	Ophthalmologist	Prospective	A novel validation tool was developed and proposed to support ophthalmologists to identifying patients at risk of falling at routine visits. Predictors of prospective falls included recent vision decline, and protective factors included use of prescribed spectacles and cataract surgery.
Miyawaki *et al*., 2012	USA	Optometrist	Cross‐sectional	Explored the potential for optometrists to refer older adults to exercise programs for falls prevention. 90% of patients said they would follow exercise referral from optometrist, and 97% of optometrist indicated they are willing to prescribe exercise programs to patients.
Shader, 2019	USA	All	Editor‐in‐chief note	Suggests that visual problems do not receive attention in falls research, and reviews the literature on visual impacts that can contribute to falls risk.
Usmani *et al*., 2021	USA	Ophthalmologist	Retrospective cohort	Examined the epidemiology of eye trauma as a result of falls in national emergency departments. Older adults were more likely to have eye trauma than adults in the setting of falls. Concluded that ophthalmologists should develop guidelines for recognizing and counseling groups at risk, and develop strategies for the prevention of fall‐related eye trauma.
Yang *et al*., 2012	UK	All	Conference abstract of cross‐sectional	Surveyed patients referred to a falls clinic and, by an optometrist, a low‐vision clinic. Although one‐third of falls clinic patients had low vision, only 7% were seen at the low‐vision clinic, suggesting the need for increased awareness and better integration between clinics.

### Falls risk stratification

Most articles (*n* = 11) discussed falls screening or risk stratification.^11^, ^33^, ^34^, ^35^, ^36^, ^37^, ^38^, ^39^, ^40^, ^41^, ^42^ Although one study found that most eye care practitioners are aware of the link between vision and falls,^34^ some studies implied that eye care practitioners might need more awareness of falls risk.^36^, ^37^, ^38^, ^39^ Many studies suggested that practitioners might not be asking about a history of falls,^39^, ^40^, ^41^, ^42^ and might not be carrying out risk stratification.^11^, ^34^, ^35^ For example, a survey of optometrists found that 81% did not assess falls risk in routine examinations.^34^ One study suggested that optometrists are aware that their patients might not be wearing the correct spectacle prescription for specific tasks,^35^ but whether this information informed subsequent assessment and management decisions was unclear. Another study suggested that ophthalmologists might not be recognizing the high‐risk nature of patients who present with fall‐related eye trauma, and that guidelines are required for counseling and strategies to prevent future falls.^11^

### Assessment

Only two sources provided insight into visual assessment for falls prevention.^38^, ^43^ An editor‐in‐chief note suggested that stereopsis is not routinely tested in older patients, as the author had specifically requested their optometrist to test their depth perception.^43^ No studies reported on eye care practitioners’ awareness or carrying out contrast sensitivity testing in the context of falls. One source suggested that greater importance might need to be placed on the link between visual fields among eye care practitioners, especially inferior field loss and its link to falls.^38^ However, it was unclear whether eye care practitioners are screening for visual field loss in older adults.

### Management

Management relating to falls prevention was reported in most articles (*n* = 17).^11^, ^31^, ^32^, ^33^, ^34^, ^35^, ^36^, ^37^, ^38^, ^39^, ^40^, ^41^, ^42^, ^44^, ^45^, ^46^, ^47^ For exercise and environmental interventions, many articles revealed a difference between theoretical and practical uptake. For example, in an optometrist survey, few optometrists referred patients to exercise programs, but the majority (97%) indicated they were willing to prescribe community‐based exercise programs for patients.^34^ Similarly, a Delphi panel of optometrists agreed that advising patients about home modification was important, but few actually did so in practice.^40^ Possible reasons for this were that optometrists were unfamiliar with referral pathways to exercise programs and other allied health professionals.^40^ A qualitative study found that ophthalmologists refer patients to practitioners, such as neurologists, but there was confusion regarding which practitioners are most helpful for falls.^44^ There were also inconsistencies in which practitioner patients were referred to.^44^ Another study suggested that eye care practitioners might not be providing advice to patients with visual impairment on strategies to prevent tripping and other incidents at home.^39^

For vision interventions, it was emphasized in one review that eye care practitioners might need more awareness on the benefits of cataract surgery for falls prevention.^37^ It was also implied that eye care practitioners might benefit from more education regarding preventing falls after cataract surgery due to magnification changes,^40^ and that it is the responsibility of the practitioner to educate the patient.^33^, ^45^ In the context of multifocal and bifocal spectacles, some studies suggested that optometrists are aware of the problems caused by such spectacles, and that they provide advice to patients on how to wear them.^31^ However, a review suggested that practitioners have insufficient awareness of the link between multifocal use and falls‐related injuries.^36^ Despite some awareness of the evidence, practitioners continue to prescribe multifocal spectacles,^36^ with optometrists in a Delphi study expressing hesitation with recommending single‐vision spectacles outdoors due to financial and non‐compliance factors.^40^

Optometrists on a Delphi panel said they would refer patients to occupational therapy and low‐vision clinics, but actual rates in practice were low.^40^ Similarly, it was implied that ophthalmologists might not be directing patients to low‐vision services.^46^ One behavior reported to be undertaken by optometrists was to avoid prescribing large changes when modifying a spectacle prescription to facilitate adaptation.^35^, ^45^ It was suggested, however, that this might be in response to patient feedback rather than due to awareness of falls evidence.^35^, ^45^ Optometrists were willing to prescribe a large change in spectacle correction if the patient was involved in decision‐making, despite the potential increased risk of falls.^40^ However, the panelists acknowledged that they would discuss the risks associated with this decision with the patient.^40^

### Barriers and enablers

Few studies reported on barriers and enablers. Time constraints was reported as a barrier for detailed fall‐related history taking^40^ and referral to exercise programs.^6^, ^34^ Other reported barriers for referring patients to exercise programs included lack of training and awareness, and unfamiliarity with falls risk assessments.^34^ There were also concerns expressed by optometrists around potential inappropriate referral to low‐vision clinics and inadvertently increasing a fear of vision loss for patients.^40^ A suggested enabler for falls prevention was to incorporate electronic record card prompts to remind clinicians of recommendations, such as using single‐vision distance spectacles for outdoor activities.^40^

## Discussion

The present scoping review aimed to map the awareness, knowledge and behaviors of eye care practitioners in falls prevention. Broadly, there appeared to be a general lack of awareness and behaviors among practitioners across risk stratification, assessment and management for falls prevention. A lack of awareness suggests more education might be beneficial for eye care practitioners to better understand the evidence for falls, and realize their role in falls prevention. Visual field assessment in patients with glaucoma was not highlighted in the World Falls Guidelines,^13^ but the literature suggests that this could be an important factor in vision testing for falls prevention.^48^ In particular, glaucomatous and other types of inferior visual field loss might increase risk of falls due to the inability to see objects and hazards when walking.^49^ Therefore, eye care practitioners should consider risk stratification for patients with glaucoma.

Optometrists, often as primary eye care providers, have many opportunities for falls prevention, including falls screening for all older adults, appropriate spectacle prescription and advice, and referrals for cataract surgery. Ophthalmologists, as secondary and tertiary care providers, also have a crucial role to play, particularly with regard to ocular trauma and low‐vision referrals.^46^ Open globe injuries from a fall should immediately flag the patient as high risk of subsequent falls, presenting an opportune time for ophthalmologists to provide falls education and appropriate referral.^11^ Ophthalmologists could also identify patients at risk of falling due to changes in depth perception after cataract surgery and inform patients accordingly.^22^ The available evidence suggested that both optometrists and ophthalmologists could improve practices in falls prevention.

A key gap identified in the present review was the limited referrals from eye care practitioners to other health professionals. Contributing factors included a lack of knowledge and clarity around referral pathways, and which professionals to refer to.^40^, ^44^ Falls prevention inherently requires a multidisciplinary approach, and concerted efforts from health professionals are necessary to optimize patient outcomes.^13^ It has been acknowledged that vision is often excluded from multidisciplinary care plans, and that general practitioners rarely refer to optometrists for vision assessment as part of a falls program.^32^ Future changes to collaborative models and integration of information sharing systems between disciplines might contribute to enhancing interdisciplinary referrals.

There was limited literature reporting on enablers and barriers to falls prevention by eye care practitioners. A meta‐synthesis on exercise recommendations for falls prevention involving general practitioners, medical specialists, nurses, allied health and other health personnel found that time constraints was a reported barrier.^50^ It was not specified whether any optometrists or ophthalmologists participated in this study.^50^ A qualitative study on exercise promotion by eye care practitioners, which included optometrists and ophthalmologists, found barriers included time constraints, a lack of knowledge, safety concerns, fear of negative experiences and resistance from patients.^6^ The study found facilitators included written resources and organizational change, which referred to redefining expectations of eye care practitioners and collaborations with other health professionals.^6^ Although this study did not focus on exercise for falls prevention, many of the findings could be applicable to exercise program referrals for falls.

Research translation refers to the process whereby evidence‐based guidelines are implemented to practice.^51^ Evidence‐based falls guidelines have been developed for optometrists in Australia,^52^ Europe^52^ and the UK,^53^ but the implementation rates of these are unknown. Falls prevention research might poorly translate to real‐world situations due to several factors. An example from the present study was the reluctance of optometrists to prescribe single‐vision distance spectacles for outdoor wear, despite being aware of the evidence.^40^ Clinicians reported reasons, such as patient preferences, cost and convenience,^40^ underscoring the crucial role of patients in the process of shared decision‐making.^54^ Beyond clinical evidence, it is important to consider factors, such as context and individual needs, for implementation. In some cases, alternative options, such as prescribing a lower addition in multifocal spectacles^35^ or providing advice on head‐gaze,^55^ might need to be explored.

A limitation of this scoping review was that only three studies directly explored the perceptions of practitioners.^34^, ^40^, ^44^ Future studies could explore the views and behaviors of eye care practitioners to better understand self‐reported awareness and implementation of falls evidence. Results could facilitate the identification of gaps in practice and inform targeted strategies to improve patient outcomes. This study only included sources from Australia, the USA, the UK and Italy. Only including studies published in English could have contributed to an absence of studies from other higher‐income countries. Further studies involving the patient perspective could also provide insight into research translation of guidelines for shared decision‐making. Finally, implementation studies would be useful to assess falls prevention interventions and their impact on clinical practice.

The present scoping review found that although eye care practitioners possess some level of awareness regarding falls prevention, many opportunities exist for further education, especially in the areas of risk stratification and management. This study identified gaps between eye care practitioners' falls prevention knowledge and implementation. Future studies should focus on identifying specific barriers and enablers to eye care practitioners implementing evidence‐based falls management into their community practice. By addressing these gaps, eye care practitioners have the potential to make a substantial impact on falls prevention, and to improve safety and quality of life of older adults.

## Disclosure statement

The authors declare no conflict of interest.

## Author contributions

AMH and KA designed the study. All authors contributed to data extraction and mapping. JC drafted the manuscript. All authors critically reviewed the manuscript, contributed to writing and approved the final manuscript.

## Ethics statement

Ethics approval was not required for this scoping review.

## Supporting information


**Data S1.** Supplementary File S1.

**Data S2.** Supplementary File S2.

## Data Availability

Data sharing is not applicable to this article as no new data were created or analyzed in this study.
