# Supplementary material for: Eye care practitioners and falls prevention for older adults: A scoping review
Source: Geriatr Gerontol Int. 2025 Feb 10;25(3):337–45. doi: 10.1111/ggi.15098 (PMC11911153; doi:10.1111/ggi.15098)
Supplement: Supplementary file 1 — Data S1. Supplementary File S1. [file GGI-25-337-s001.docx]

**Supplementary File 1**

MEDLINE Literature Example Search Strategy

Database: Ovid MEDLINE(R), Ovid MEDLINE(R) In-Process & Other Non-Indexed Citations, Ovid MEDLINE(R) Daily and Ovid OLDMEDLINE(R) <1990 to Present>

Search Strategy:

1. (optometr* or optic* or ophthalmolog* or eyecare professional or eyecare practitioner or eye care profession* or eye care practitioner).tw.
2. Optometrists/ or optometry/ or ophthalmology/ or Ophthalmologists/

3. 1 or 2

4. fall*.tw.

5. Accidental Falls/

6. 4 or 5

7. exp aged/ or exp geriatrics/ or exp geriatric nursing/ or (centarian* or centenarian* or elder* or eldest or frail* or geriatri* or nonagenarian* or octagenarian* or octogenarian* or old age* or older adult* or older age* or older female* or older male* or older man or older men or older patient* or older people or older person* or older population or older subject* or older woman or older women or oldest old* or senior* or senium or septuagenarian* or supercentenarian* or very old*).tw.

8. Aged/ or "Aged, 80 and over"/

9. 7 or 8

10. 3 and 6 and 9
