# Supplementary material for: Eye care practitioners and falls prevention for older adults: A scoping review
Source: Geriatr Gerontol Int. 2025 Feb 10;25(3):337–45. doi: 10.1111/ggi.15098 (PMC11911153; doi:10.1111/ggi.15098)
Supplement: Supplementary file 2 — Data S2. Supplementary File S2. [file GGI-25-337-s002.docx]

**Supplementary File 2**

**Table 1: Fall risk stratification and assessment evidence mapped against the World Falls Guidelines (WFG) recommendations**

| **(i) Falls risk stratification and algorithm** | | |
| --- | --- | --- |
| **WFG Recommendation (Montero-Odasso et al. 2022)** | **Theme** | **Illustrative quote(s)** |
| Older adults at low risk for falls should be offered education about falls prevention and exercise  Older adults at intermediate risk should be offered targeted exercise or a physiotherapist referral in addition to the above  Older adults at high risk should be offered a multifactorial falls risk assessment to inform individualised tailored interventions | ECP may not performing risk stratification (Elliott, Kavoussi et al., Usmani et al., Miyawaki et al.) | “Although the majority (90%) [of optometrists] believed in a vision-fall connection, 81% of them did not assess patients’ fall risks in their routine examination.” (Miyawaki et al.) |
|  | ECP are aware that patients do not wear the correct spectacles when they should (Elliott) | “and it has been shown in accident research (and is well known to optometrists) that people do not always wear their spectacles when they should and can even wear reading glasses when walking about.” (Elliott) |
|  | Most ECP are aware of falls risk and visual impairment (Miyawaki et al.) | “Most were aware of evidence linking older adults’ vision and falls” (Miyawaki et al.) |
|  | ECP may need more awareness of the link between multifocal glasses and falls risk (Chang), as well as visual impairment and falls (Black & Wood, Black et al., Boon et al.) | “Furthermore, the cases of falls related to multifocal spectacles is likely to be under-reported or missed, given that eye care providers and patients themselves may not be aware of the direct link between multifocal spectacles and falls.” (Chang)  “…impaired visual function… can approximately double the risk of a fall. This is an important association that warrants higher levels of awareness” (Black & Wood) |
| **Opportunistic case-finding**  Clinicians should routinely ask about falls in their interactions with older adults, as they often will not be spontaneously reported  Older adults in contact with healthcare for any reason should be asked, at least once yearly, if they have experienced falls in the last 12 months and details about the fall/s | ECP may not be asking patients about falls in history taking (Boon et al., Elliott, Ho et al., Mehta et al., Garrigan et al.) | “Recommendations that reached consensus but had low uptake in the panelists' practices were history taking about the risk of falls” (Ho et al.) |
| **Older adults presenting with falls or related injuries**  Should be asked about the details of the event and its consequences  Should be regarded as high risk of future falls | ECP may not be recognising risk and prevention of falls for those presenting with fall-related eye trauma (Usmani et al.) | “It is important to recognise such differences in populations that experience eye trauma, particularly in the event of a fall, which will allow better recognition of at-risk groups by ophthalmologists, more directed and focussed counselling to enable prevention of falls and associated eye-trauma.” (Usmani et al.) |
| **(ii) Vision and hearing assessment** | | |
| **WFG Recommendation (ref)** | **Theme** | **Illustrative quote(s)** |
| Enquire about vision impairment as part of a multifactorial risk assessment, measure visual acuity and examine for other visual impairments such as hemianopia and neglect when appropriate | The importance of visual fields and link to falls tends to be overlooked by ECP (Black et al.) | “the findings of this study and that of other recent studies highlight the importance of screening for visual field loss as an integral component in falls risk assessments” (Black et al.) |
| Visual screening should not be limited to measurement of visual acuity and should incorporate contrast sensitivity and depth perception | ECP are not routinely testing depth perception (Shader) | “When I went for my next cataract checkup with my optometrist, I asked that my depth perception be tested; she found it to be impaired.” (Shader) |

**Table 2: Management and interventions evidence mapped against the World Falls Guidelines (WFG) recommendations**

| **WFG Recommendation** | **Theme** | **Illustrative quote(s)** |
| --- | --- | --- |
| **Exercise interventions** | | |
| Exercise programmes for fall prevention for community-dwelling older adults should be offered | ECP should provide education and referral on exercise but few ECP are doing so (Miyawaki et al.) | Since 95% of the patients in this study believed there is a connection between vision and falls, during an eye examination, optometrists could leverage this belief and further inform patients about the associated benefit of exercise to reduce falls” (Miyawaki et al.)  “In spite of the positive attitudes toward fall prevention and exercise program referrals from optometrists and their patients, few optometrists … referred patients to any community-based program.” (Miyawaki et al.) |
|  | ECP may not be familiar with direct referral pathways to other professions and community programs (Ho et al.) | “a multidisciplinary approach is recommended to prevent falls through interprofessional referral pathways or community programs but was not well known to the optometrists on this panel.” (Ho et al.)  “Referral to general practitioners was  also suggested because general practitioners could refer the patients  to respective networks including physiotherapists and orthopedic  clinics.” (Ho et al.) |
| **Environmental interventions** | | |
| Recommendations for modification of an older adult’s physical home environment for fall hazards should be provided by a trained clinician | ECP say they would refer patients at risk of falling to low vision clinics for home modification but may not be doing so in practice (Ho et al.) | “[Panelists] would advise patients with visual impairment who were at moderate to high risk of falling for home modifications to prevent falls via other channels including low-vision clinics or community organizations providing low-vision rehabilitation services like Vision Australia and Guide Dogs Australia. (Ho et al.)  “Recommendations that reached consensus but had low uptake in the panelists' practices were … provision of advice to patients to seek home modification via occupational therapists and other professionals.” (Ho et al.) |
|  | ECP may not be providing patients with visual impairment with advice for how to avoid home incidents, including tripping (Boon et al.) | “Although many participants were able to identify a near miss or accident and the causative factors, they were often unable to think of preventative factors. Examples of incidents AMD participants reported that they had no ideas about how to prevent include the following: …misjudging distance when closing a door, and missing/tripping on steps.” (Boon et al.)  “Most participants guessed at strategies that they thought might help, suggesting that they did not recall appropriate advice from their eye care providers, who have the expertise to provide this kind of advice.” (Boon et al.) |
| **Vision interventions** | | |
| Cataract surgery for the first eye and for both eyes | ECP may need more awareness of link between cataract and falls risk (Black & Wood) | “Optometrists need to be aware… [falls can be reduced following cataract surgery]” (Black & Wood) |
|  | ECP may need more education regarding post-cataract falls prevention (Ho et al.) | “Another suggestion to improve the implementation of [falls prevention] recommendations was a provision of education to optometrists on fall prevention for patients after cataract surgery.” (Ho et al.) |
|  | Patients may need more awareness on post-cataract, which suggests ECP may not be providing it (Kavoussi et al.) | “Because visual loss is not a commonly discussed consequence of falls, it is incumbent upon the ophthalmologist, who deals primarily with the geriatric population, to contribute to the discussion of fall risk and direct the pseudophakic patient to appropriate educational resources.” (Kavoussi et al.)  “Certainly, the magniﬁcation effects of changing spectacles and having cataract or refractive surgery focus on the positive effect on visual acuity with myopia reduction, and previously there has been no thought to the effect of ocular or spectacle magniﬁcation on mobility and falls.” (Elliott & Chapman) |
| Older adults should avoid wearing multifocal glasses when outside | ECP and patients need more awareness regarding link between falls risk and multifocal glasses (Chang) | “… patients who fall typically seek treatment for their fall related  injuries without considering the influence of their multifocal spectacles. Therefore, the link between a chronic strategy of presbyopia correction and the acute injuries associated with that strategy remains elusive to eye care providers” (Chang) |
|  | ECP are aware of the problems that can be caused by multifocal glasses (Hill et al.) | “Some participants reported that their Optician or Optometrist had warned  them of problems [of multifocal spectacles]” (Hill et al.) |
|  | ECP may not be encouraging patients to wear single vision lenses (Black & Wood) and are continuing to prescribe multifocal glasses (Chang) | “For example, optometrists should promote the use of single vision lenses for  walking in patients who are high-risk fallers...” (Black & Wood)  “Despite evidence in the literature that multifocal spectacles and monovision substantially increase the risk for falls in the older population, many eye care providers continue to use these strategies for the correction of presbyopia.” (Chang) |
|  | ECP are hesitant to recommend single vision glasses for outdoors (Ho et al.) | “Panelists commented that optometrists hesitated to change the wearing habit of patients who were proficient wearers of bifocals/progressive addition lenses to additional single-vision glasses because of the likely noncompliance and financial disincentives to the patients to have an additional pair of glasses for walking” (Ho et al.) |
|  | ECP provide advice to patients about adaptive behaviours whilst wearing multifocal spectacles (Hill et al.) | “Some participants reported that their Optician or Optometrist had warned them of problems and had given advice such as: "Don’t be afraid to show your double chin." (Hill et al.) |
| Occupational therapy interventions involving home hazard reductions are effective in preventing falls in older adults with severe visual impairments | ECP are aware of the ability to refer to occupational therapy (Ho et al.), have an opportunity to refer to occupational therapy and low vision clinics (Usmani et al.) but appear to have limited awareness (Ho et al., Miyawaki et al.) | “At least four panelists suggested that an occupational therapist was the professional to refer to…Furthermore, panelists commented that most low vision  clinics have an associated occupational therapist.” (Ho et al.)  “Recommendations that reached consensus but had low uptake in the panelists' practices were … provision of advice to patients to seek home modification via occupational therapists and other professionals.” (Ho et al.)  “They would advise patients with visual impairment who were at moderate to high risk of falling for home modifications to  prevent falls via other channels including low-vision clinics or community organizations providing low-vision rehabilitation services like Vision Australia and Guide Dogs Australia. (Ho et al.)  “Ophthalmologists may therefore have an opportunity to reduce the incidence of falls through interventions such as early referral to low vision services and occupational therapy.” (Usmani et al.) |
|  | ECP may not be referring to low vision services (Evans & Rowlands) | “Even the cases where pathology is diagnosed and who are seen by an ophthalmologist often fail to receive appropriate low vision services.” (Evans & Rowlands) |
| It is recommended that optometrists counsel their clients about likely short-term increased fall risk when dispensing new prescription glasses | Some optometrists prescribe partial prescriptions, although not due to falls evidence but due to feedback from clinical experience (Elliott & Chapman, Elliott) | “Clinicians suggest that adapting to new spectacles is more difficult for older adults, and it is certainly a major concern for older patients attending an eye examination. For these reasons, some clinicians recommend only prescribing partial changes in refractive error to help adaptation, particularly in older patients.”(Elliott & Chapman)  ‘Unfortunately, these recommendations are not supported by any research evidence (they are based on clinical experience gained from dissatisfied patients who return to complain about their spectacles) and do not appear to be widely used” (Elliott & Chapman)  “Although experienced optometrists in the United Kingdom have reported that they ‘‘partially prescribe’’ in response to a questionnaire containing a selection of clinical vignettes, less experienced optometrists more commonly prescribe the full subjective refraction result. This suggests a need for further education and training in this area of clinical practice. Patients should also be warned of magnification changes with new spectacles…” (Elliott) |
|  | ECP would be willing to prescribe large amounts of prescription changes (even if risk of falls may increase) if the patient was involved in decision making (Ho et al.) | “If the change in spectacle correction was bigger than recommended for a patient at risk of falls (i.e., >0.75 D), around threequarters of panelists (8 of 11 [73%]) agreed that patients must be involved in the decision making to update spectacle correction. Although nearly two-thirds of panelists (7 of 11 [64%]) agreed to  prescribing/updating the spectacle correction, which could increase the risk of falling when patients were involved in decision making, the panelists stressed the need for risk analysis with proper communication (e.g., gradually building of wearing time) between all parties.” (Ho et al.)  “the panelists have mentioned that patients need to be involved in decision making and risks should be discussed when a change in spectacle correction exceeds the recommended range.” (Ho et al.) |
|  | ECP may not be informing their patients regarding magnification changes and falls risk (Elliott & Chapman, Elliott) | “In addition, all older patients should be appropriately warned of the effects of changed refractive error after cataract surgery and/or when they first receive new spectacles on the apparent position and size of steps and stairs” (Elliott & Chapman) |

**Table 3: Barriers and enablers evidence**

|  | **Theme** | **Illustrative quote(s)** |
| --- | --- | --- |
| **Barriers** | Optometrists anticipate non-compliance due to factors such as convenience and financial disincentive (Ho et al.) | “Panelists commented that optometrists hesitated to change the wearing habit of patients who were proficient wearers of bifocals/progressive addition lenses to additional single-vision glasses because of the likely noncompliance and financial disincentives to the patients to have an additional pair of glasses for walking.” (Ho et al.) |
|  | Time constraint is a barrier for history taking (Ho et al.) | “Previous studies have shown that the more risk factors an older adult has, the more likely he/she will fall, so a detailed history is important to analyze the risk of falls of a patient. However, time constraint has been reported as a key barrier in detailed history taking for Australian optometrists. (Ho et al.) |
|  | Unfamiliarity with fall assessments and lack of training is more of a barrier than time and reimbursement (Miyawaki et al.) | “Although the majority (90%) believed in a vision-fall connection, 81% of them did not assess patients’ fall risks in their routine examination. The reasons were because fall risk assessment is not necessary/required (23.9%), they were not trained for it (19.6%), they had never thought of it (18.7%), there is no time for it (14.3%), and there is no reimbursement for it (2.6%).” (Miyawaki et al.)  “In spite of the positive attitudes toward fall prevention and exercise program referrals from optometrists and their patients, few optometrists conducted fall-risk assessments as routine vision care or referred patients to any community-based program. It is noteworthy that the lack of reimbursement was not a barrier to conducting fall risk assessments, as only 2.6% of the optometrists indicated that no funding was a factor. Rather, our findings suggest that other factors, such as being unfamiliar with community-based exercise programs and fall assessments, were more salient barriers to optometrists incorporating fall-prevention efforts in their practices.” (Miyawaki et al.) |
|  | ECP have concerns about the impact of referrals to low vision services on mental health (Ho et al.) | “Panelists worried that inappropriate referral to low-vision clinics might impact patients' mental health and add to fear of losing vision” (Ho et al.) |
| **Enablers** | Reminders may help optometrists consider recommending wearing single vision spectacles outside (Ho et al.) | “However, because the literature strongly supported this recommendation [of single vision distance spectacles outdoors], panelists suggested adding a prompting reminder in the electronic records system for optometrists to consider this recommendation on a case-by-case basis” (Ho et al.) |
